# Effect of Freckle Defects on Hot Deformation Behavior and Dynamic Recrystallization Structure Inheritance of an Iron–Nickel-Based Superalloy

**DOI:** 10.3390/ma19061113

**Published:** 2026-03-13

**Authors:** Lianjie Zhang, Xiaojia Wang, Yuhan Wang, Lei Wang, Ran Duan, Shuo Huang, Guohua Xu, Yang Liu

**Affiliations:** 1 Gaona Aero Material Co., Ltd., Beijing 100081, China; zhanglianjie26@126.com (L.Z.);; 2 The Key Laboratory for Anisotropy and Texture of Materials (Ministry of Education), School of Materials Science and Engineering, Northeastern University, Shenyang 110819, China

**Keywords:** GH4706 alloy, freckle defects, thermal deformation behavior, hot processing map, dynamic recrystallization

## Abstract

To study the influence of freckle defects on the hot deformation behavior and the inheritance of dynamic recrystallization (DRX) structure in GH4706 alloy, the microstructures of specimens with and without freckles and the evolution laws of hot-processing parameters were compared. Hot compression experiments were conducted on a thermal simulation testing machine at 950–1150 °C, strain rates of 0.001–1 s^−1^, and 55% deformation. Freckle-containing specimens were tested under DRX critical conditions. The flow stresses of both specimens increase with strain rate or with decreasing temperature. The power dissipation coefficient (η) and instability value (ξ) follow complex laws. Electron back-scattering diffraction (EBSD) was used to analyze DRX microstructures and nucleation mechanisms. The DRX degree of freckle-containing specimens is lower, with a larger average grain size. The DRX mechanism initiates preferentially in freckle-containing specimens, and its volume fraction changes in a complex manner. Grain coarsening occurs in freckle-containing specimens at high temperatures and low strain rates. Freckle defects lead to significant differences in the DRX mechanism of GH4706 alloy. Freckle-containing specimens exhibit both discontinuous dynamic recrystallization (DDRX) and continuous dynamic recrystallization (CDRX), whereas freckle-free specimens primarily display DDRX and second-phase particle-stimulated nucleation (PSN). The presence of MC carbides and Laves phases within freckle defects provides nucleation sites, further supporting a typical second-phase particle-stimulated nucleation mechanism.

## 1. Introduction

A new age-hardening alloy, GH4706, reinforced by Nb and Ti, is a superalloy based on Fe, Ni, and C [1,2]. It is frequently used in large forgings such as turbine discs and shafts for aircraft engines and marine gas turbines due to its stable microstructure, long high-temperature fatigue life, and exceptional flexibility. Because of its excellent castability, machinability, and consistent high-temperature mechanical properties, GH4706 alloy can be used to produce large disc forgings exceeding 2000 mm in diameter [3,4,5]. Now, GH4706 alloy is primarily used to make turbine discs for heavy-duty gas turbines of the “E” and “F” classes [6,7]. However, when producing massive turbine discs, metallurgical defects (freckles) remain an unavoidable problem [8,9,10,11,12,13,14]. Ren [15] examined the underlying mechanism of freckle development, attributing it to convection channels associated with hot solute transport in the intermediate region during solidification. This mechanism is driven by density changes generated by inter-dendritic segregation. In vacuum arc remelted (VAR) and electroslag remelted (ESR) ingots, the formation mechanism of freckles mainly consists of four stages: First, the high cooling rate in the near-wall region leads to overgrowth of dendrites and forms channel segregation next to dendrites; secondly, the enriched solute is transferred to the near-wall solid–liquid interface to overheat the local liquid metal, and the top of the dendrite stem melts and detaches to form isolated fragments. Third, the downstream side of planktonic fragments continues to grow, the upstream side of the settled fragments is melted, and new tiny fragments form on the side branches. Finally, the supercooling degree of the cooling process makes the channel fragments grow into equiaxial crystals, and after the side branches block the channel, as the growth orientation of the original dendrites aligns with the <001> preferred direction, the original dendrites grow more rapidly and eventually envelop the equiaxed grains. Consequently, freckles typically form at the R/2 area of the ingot.

Freckles, as a local segregation region of microscopic chemical composition and grain structure, can easily become the starting point of stress concentration under high-temperature cyclic loading, which may weaken the mechanical properties of the material at high temperatures. The grain boundary region formed by Freckles has different chemical compositions and strengthening phase distributions (such as γ’ and γ” phases), which weakens the precipitation strengthening effect at high temperature and makes the material more prone to creep deformation under high-temperature cyclic load. At the same time, the stress concentration effect in the Freckles region leads to the fixed location of crack initiation and the obstruction of the crack propagation path [16,17]. In addition, for electrochemical behavior, component segregation in the Freckles region can lead to local microcell effects, making local intergranular corrosion or oxidation more likely to occur [18]. In high-temperature oxidizing environments, grain boundaries in the Freckles region tend to be the preferred growth paths for oxides. The oxide layer may grow unevenly in this area, or the oxide layer may not bind tightly to the matrix due to grain boundary diffusion, increasing the risk of oxidative delamination. Due to the discontinuity of the grain boundary structure, the Freckles region may become a rapid diffusion channel for oxide ions, which accelerates the occurrence of internal oxidation and weakens the overall durability [19]. The above mechanical and electrochemical influences pose new challenges to the application potential and need to be fully considered in process control, material selection, and evaluation systems.

The current study reveals that the combination of triplex refining (vacuum induction melting (VIM) + ESR + VAR) promotes purity and microscopic uniformity [20,21,22]. However, improper control of melting parameters, such as inappropriate ESR current, voltage, or melting rate, may lead to molten-pool instability, resulting in element segregation or inclusion enrichment. Variations in the material’s melting rate produce temporary changes in the ingot growth rate and the thermal gradient in the slug zone, leading to melt-related defects. These defects may subsequently result in freckles during subsequent processing. Furthermore, the probability of metallurgical defects in superalloy is highly related to the morphology of the molten pool during VAR and the solidification behavior of the ingot [21]. Reducing element segregation is another favorable outcome of the VAR process. However, due to delayed electrode melting and the limited molten-pool area, uniform element dispersion can be achieved only locally. Specific metallurgical defects (freckles and foreign inclusions) tend to concentrate on locations of the VAR ingot [23]. Furthermore, Yang and colleagues [24] adopted a mixed strategy combining numerical simulation and industrial experimentation. Using the Rayleigh model, Yang computed changes in the electromagnetic, flow, and temperature fields, as well as the shape of the molten pool, throughout the entire VAR melting process for a 660 mm GH4742 superalloy ingot. Yang’s findings were validated by comparing the projected sites of freckle defects with the observed positions of defects in the ingot structure and element segregation patterns. It demonstrates that fine control of solidification kinetics factors can enhance ingot metallurgical quality and potentially minimize macroscopic segregation faults in large-sized IN706 ingots. Huang [25] developed a VAR ingot molten pool depth controller to predict molten pool depth, although differences from actual depths necessitate more quantitative validation through microscopic characterization.

Techniques for eliminating metallurgical defects, such as freckle defects, are still in the early stages of development. Freckle defects significantly degrade alloy performance, as freckle defects cannot be eliminated by post-casting thermal–mechanical treatment and may even be inherited into the forging process [26]. The existing literature mostly focuses on the formation mechanism of freckles during smelting and casting, as well as the optimization of their microstructure. There is still a lack of systematic and quantitative research on the improvement of hot working performance and the influence of segregation elements on its performance, as well as the microstructure inheritance issue of freckles during the subsequent hot deformation process and the specific impact of this inheritance on the evolution of DRX mechanism, grain evolution and the distribution of the second phase, especially in triplex. To fill this gap, this study will systematically reveal the inheritance mechanism of freckles in GH4706 alloy and its influence on the microstructure of thermal processing under the refining (VIM + ESR + VAR) system.

## 2. Experimental Materials and Research Methods

**Figure 1:**
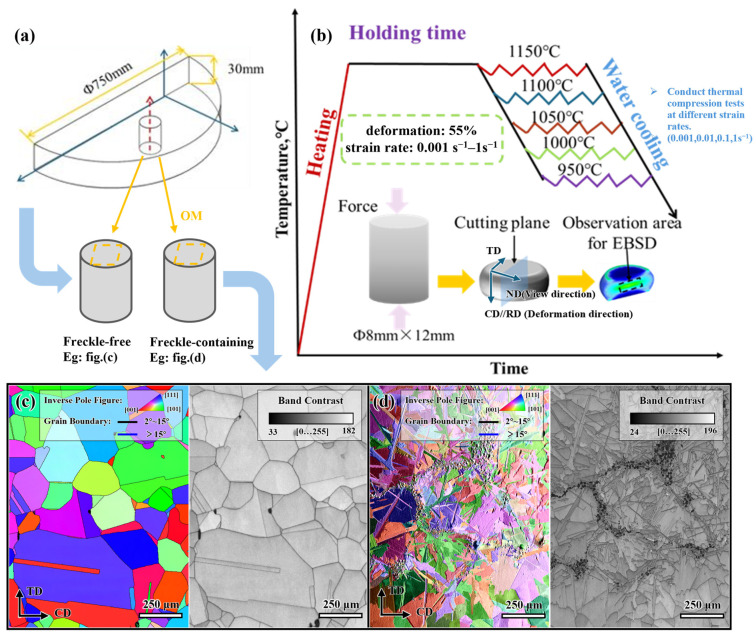
Initial microstructure characterization of as-forged GH4706 alloy and schematic of thermo-mechanical processing: (**a**) Specimens sampling diagram: Sampling is performed on the turbine disc workpiece at a radius equal to the center, and freckled and non-freckled are distinguished in turn. The red arrow indicates the sampling position, and the yellow arrow indicates the sample type determined by OM; (**b**) Thermal deformation process diagram: First, heat the alloy to 1150 °C and hold it for 15 min. Then, for 20 groups of specimens (each group containing 2 freckle-containing specimens and 2 freckle-free specimens), cool them to 950, 1000, 1050, 1100, and 1150 °C, respectively, and conduct hot compression under the conditions of 0.001, 0.01, 0.1, and 1 s^−1^ (a total of 20 compression conditions). Control the compression process within 0.5 min, and then quickly quench them in water. Prepare the longitudinal section of the alloy for EBSD analysis; (**c**) Inverse Pole Figure (IPF) and Band Contrast micrograph of freckle-free specimens; (**d**) IPF and Band Contrast micrograph of freckle-containing specimens.

The experimental material was a GH4706 alloy turbine disc measuring Φ750 mm × 30 mm, produced using a process involving “triplex refining + homogenization + repeated upset-draw cogging” (elemental composition wt.%: Al-0.326, Ti-1.87, Nb-3.14, Cr-15.7, Ni-43.1, Fe as balance). The forged GH4706 alloy specimens with and without freckles were cut from the disc with the same radius shown in Figure 1a. The positions of freckle defects were confirmed by non-destructive testing (ultrasonic flaw detection). The size of the specimens is Φ8 mm × 12 mm, and a total of 20 groups of specimens were cut, each containing 2 freckle-containing specimens and 2 freckle-free specimens. In order to avoid the confusion of the freckle effect caused by the uneven grain size and columnar structure caused by the position of the ingot, a group of freckle-containing and freckle-free specimens with similar grain size and columnar structure distribution was selected to compare the effect of highlighting the freckle.

An optical microscope (OM) was used to verify and observe the freckle-containing specimens and freckle-free specimens before thermal compression. Only select the specimens with uniform freckle size and distribution to ensure the consistency of experimental conditions. The specimen observation surface is ground and mechanically polished with sandpaper of different degrees of roughness until the surface of the specimen is flat and bright without other scratches, and then the specimen is chemically corroded. After corrosion, the microstructure before thermal deformation was observed under the OLYMPUS GX71 optical microscope (Olympus Corporation, Tokyo, Japan) to distinguish freckle-containing specimens from freckle-free specimens.

Before thermal deformation, the surfaces of all specimens were mechanically polished. A single-pass thermal simulation compression test with a deformation amount of 55% was carried out on a Gleeble-3800 thermal simulation testing machine (Dynamic Systems Inc., Poestenkill, NY, USA). The Gleeble tests were conducted in accordance with the ASTM E209-00(2010) [27] standard for hot compression testing of metallic materials. The critical temperature of dynamic recrystallization of GH4706 alloy is about 975 °C, and the temperature range needs to cover 950–1150 °C to study the microstructure evolution at different temperatures. When the deformation temperature is lower than the critical temperature, the alloy deformation promotes dynamic recrystallization, and the grain size decreases with the increase in deformation η. The temperature is higher than the critical temperature. Dynamic recrystallization is more likely to occur, but the upper limit of temperature (≤1150 °C) needs to be controlled to avoid excessive coarseness of the grains. Hence, the deformation temperature range is 950–1150 °C (950, 1000, 1050, 1100, 1150 °C) according to the range of alloy recrystallization conditions. Considering that the strain rate directly affects the degree of dynamic recrystallization, the low strain rate (0.001–0.01 s^−1^) provides sufficient time to promote dynamic recrystallization, which can avoid rheological instability, and the high strain rate (0.1–1 s^−1^) combined with temperature control can avoid cracking of the specimen due to excessive deformation resistance. The strain rate range is 0.001–1 s^−1^ (0.001, 0.01, 0.1, 1 s^−1^). By adjusting the combination of deformation temperature and deformation rate, a balance between dynamic recrystallization and grain refinement is achieved, and a total of 20 deformation conditions are realized, as shown in Figure 1b. Before the thermal compression, the specimens were heated to 1150 °C at a heating rate of 10 °C/s and held at this temperature for 15 min to stabilize the temperature field of the specimens. Then, 20 groups of specimens were cooled to their respective target temperatures at a rate of 10 °C/s for thermal compression experiments at different strain rates. The single compression deformation process of all specimens is completed within 0.5 min. After thermal deformation, the samples were rapidly cooled by water cooling to preserve the deformed structure.

The microscopic morphology of the material was characterized by scanning electron microscopy (SEM) of the JSM-7800F model (JEOL Ltd., Tokyo, Japan). SEM and energy-dispersive spectroscopy (EDS) mainly analyzed the freckle composition phase and elemental segregation degree of freckle-containing specimens, while EBSD mainly studied the evolution of tissue grain and grain boundary characteristics. For hot compression specimens, wire cutting is used to intercept EBSD specimens radially along the centerline of the compressed specimen, with a thickness of about 5.4 mm. The specimen was ground to 5000# with water sandpaper, then mechanically polished, and the stress layer was relieved using electropolishing. The Oxford Symmetry S2 EBSD system collected spatial orientation data at a step size of 0.1–0.5 μm and an acceleration voltage of 20 kV. The freckle-containing and freckle-free specimens of GH4706 alloys were quantitatively characterized using Aztec Crystal (2.1.2) software to examine grain boundary (GB) types, recrystallization degree, grain orientation spread (GOS) maps, and kernel average misorientation (KAM) maps, thereby investigating microstructural evolution and nucleation mechanisms. As shown in Figure 1c, the freckle-containing specimens in this study differ from the freckle-free specimens in terms of crystal orientation and microstructure. The chain structure of the freckle-containing specimens comprises a massive substructure with pronounced size differences, characterized by continuous precipitation along the original grain boundary. The small-angle grain boundaries at the interface account for about 5% of the total interface (about 2% of the matrix area) and form a local high-disorder region through the movement of nail dislocations, which promotes the accumulation of stored energy and affects the starting conditions for DRX. Although the orientation distribution of different samples is completely unavoidable, in this study, all specimens have the same grain size range (100–150 μm) and are compressed under the same cross-sectional area and total volume, showing approximately isotropic polycrystalline behavior at the macroscopic scale, so the comparison results are somewhat representative.

Based on the stress–strain curves of specimens with and without freckles under 20 different deformation temperatures and deformation rates obtained from the Gleeble-3800 thermal simulation testing machine, friction correction is carried out to reduce the errors caused by the decrease in the lubrication effect. Then, according to the Prasad standard criterion, the power dissipation coefficient (η) and instability coefficient (ξ) of samples under different conditions are calculated. The above parameters are processed using Origin (2024.lnk) software. With the strain as the distinguishing factor, 3D comparison maps of the η under different strains and thermal processing maps are drawn.

## 3. Results and Discussion

### 3.1. Characterization of Freckle Microstructure in Forged Alloy

**Figure 2:**
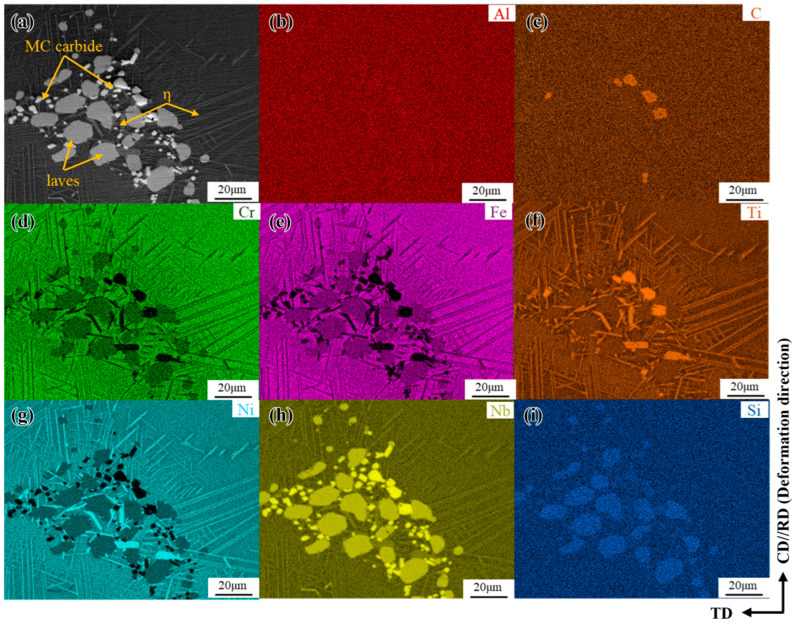
Spectrum scan analysis of the freckle composition of GH4706 alloy: (**a**) Morphology diagram, the yellow arrow points out the different phases; (**b**–**i**) Al, C, Cr, Fe, Ti, Ni, Nb, and Si EDS spectra, MC carbides mainly contain Nb, Ti and C; the η phase is primarily rich in Ni, Ti, Nb; the Laves phase is mainly enriched with Ni, Ti, Cr and Fe.

The phase-composition analysis results for the freckle defect area in the forged GH4706 alloy are shown in Figure 2. The energy dispersion spectra of Al, C, Cr, Fe, Ti, Ni, Nb, and Si of the secondary phase precipitates were scanned and analyzed by spectral scanning analysis.

The morphological characteristics and elemental distribution of the microstructure of freckles were observed by scanning electron microscopy. It is generally believed that the Laves phase is in the form of short, block-like rods, rich in Nb and Mo elements, while the η phase is mostly needle-like and plate-like, mainly enriched in Ti, Nb, and Al elements [28,29,30]. By observing and comparing Figure 2, it was found that there are small-sized granular precipitates scattered among other secondary phase precipitates, mainly enriched in Nb, Ti, and C elements, determined as MC carbide; needle-like precipitated phases were observed parallel to the grain boundaries. The EDS spectra showed that Ni, Ti, and Nb elements were mainly enriched, and it was determined to be the η phase. In addition, the massive precipitated phases are mixed and aggregated at the grain boundaries in the form of clusters with small-sized MC carbides, mainly enriching Ni, Ti, Nb, Cr, and Fe elements. At the same time, there is a small amount of mixed aggregation of oxidation inclusions rich in Si elements around the Laves phase, while Nb, Ni, and Ti show strong enrichment in the sequence Nb > Ni > Ti, C and Al are significantly depleted at the freckle defect locations. The location of the second phase is related to this elemental enrichment, indicating that the widespread precipitation of the second phase led to relative depletion or enrichment of the elements.

### 3.2. Effect of Freckle on the Flow Curve

**Figure 3:**
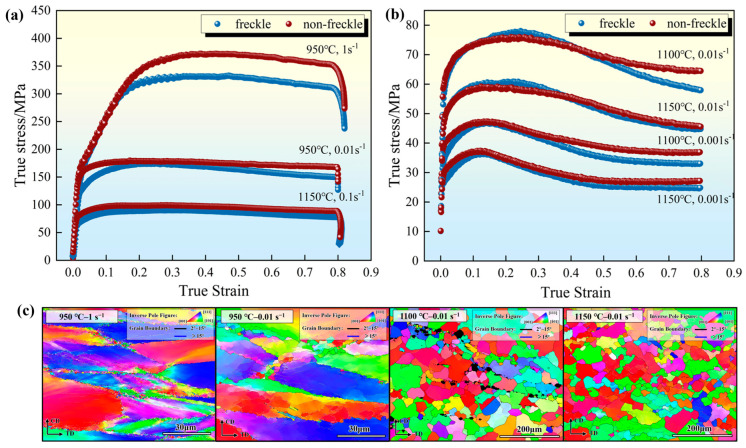
True stress–strain curves of freckle-containing specimens and freckle-free specimens of GH4706 alloy under different deformation conditions: (**a**) non-dynamic recrystallization (NDRX) zone; (**b**) DRX zone. The red line represents the test parameters of the freckle-free specimens, and the blue line represents the test parameters of the freckle-containing specimens. (**c**) The EBSD microstructures corresponding to the NDRX and DRX zones.

**Figure 4:**
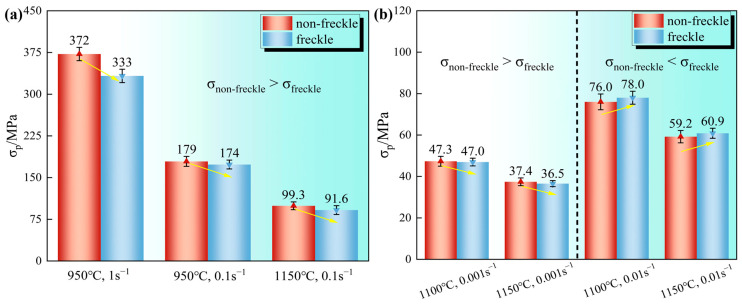
Histogram of peak stress of GH4706 alloy freckle-containing specimens and freckle-free specimens under different deformation conditions, the yellow arrow points to the changing trend: (**a**) NDRX zone: the peak stress of the freckle-containing specimens was lower than that of the freckle-free specimens; (**b**) DRX zone: at a low strain rate of 0.001 s^−1^, the peak stress of freckle-containing specimens is lower than that of freckle-free specimens, and the opposite law is reversed with the increase in strain rate. Error bars represent ±1 standard deviation from two independent experiments.

The modified stress-curve strain and peak stress of the curve for the non-freckled and freckle-containing specimens under the same deformation conditions are shown in Figure 3 and Figure 4, respectively.

Figure 3 shows the true stress-true strain curves and the corresponding microstructures over the entire compression range of freckle-free and freckle-containing specimens over multiple combined regions of deformation temperature and strain rate. The true stress and true strain values are obtained from the measured engineering data using a standard conversion formula, as shown in Equation (1) [31]:

(1) $$\sigma_{True}=\sigma_{eng}\left(1+\varepsilon_{eng}\right)\;\;\;\;\;\;\;\;\varepsilon_{True}=ln\left(1+\varepsilon_{eng}\right)$$

According to the trend of curves and their corresponding grain structure, curves are divided into NDRX zones and DRX zones. As shown in Figure 3a, under the conditions of lower temperature and higher strain rate (950 °C–1 s^−1^, 950 °C–0.001 s^−1^ and 1150 °C–0.1 s^−1^), the stress softening of the curve after peak is small and the change is not obvious, which is a non-complete dynamic recrystallization state, while the curve (1100 °C–0.01 s^−1^, 1100 °C–0.001 s^−1^, 1150 °C–0.01 s^−1^ and 1150 °C–0.001 s^−1^) in Figure 3b softens significantly after the peak and tends to stabilize with the increase in strain, which is a typical DRX characteristic [32,33]. Comparing the microstructure of the EBSD scan of the freckle-containing specimens under deformation conditions in Figure 3c, the grain size distribution in the DRX zones tends to be more homogeneous, the average grain size is smaller, and the distribution is narrower; the NDRX zones still retain more elongated grains or coarse grain boundary characteristics. The division of low-temperature and high-speed combination and high-temperature low-rate combination emphasizes the influence of temperature and deformation rate on the degree of DRX.

In the NDRX zones, the flow curves of the freckle-containing specimens were generally lower than those of the freckle-free specimens, and the difference was most obvious under the condition of low temperature and high strain rate (950 °C–1 s^−1^). In the DRX zone, an intersection between the freckle-containing and freckle-free curves was observed. With peak stress greater than that of the freckle-free specimen, the freckle-containing specimen’s flow curve first surpasses that of the freckle-free specimen and then descends below it at 0.01 s^−1^. The freckle-containing specimen’s peak stress and flow curves were both lower than those of the freckle-free specimen at a strain rate of 0.001 s^−1^. Because the freckle-containing specimen had less time to acquire energy and remove dislocations at high strain rates, dislocation pile-up was greater, thereby raising the peak stress. These observations show that freckles can affect the mechanical reaction of alloys to a certain extent, and the maximum deviation of stress may not only occur at the peak stress, but may also occur in the descending part of the curve.

### 3.3. Effect of Freckle on the Hot Deformation Behavior

A theoretical framework, the Dynamic Material Model (DMM), explains and predicts how materials change and deform at the microstructural level when heated [34,35,36,37]. The total energy (P) absorbed by the workpiece during plastic deformation can be decomposed into two parts: dissipation (G) and dissipation covariate (J), which represent the energy consumed by plastic deformation and tissue evolution, respectively, as shown in Equation (2) [38]:

(2) $$\;\sigma \cdot \dot{\varepsilon }\;=\;P\;=\;G\;+\;J\;=\int_{0}^{\dot{\varepsilon }}\sigma d\dot{\varepsilon }+\int_{0}^{\sigma }\dot{\varepsilon }d\sigma$$

At a certain deformation temperature and strain rate, the strain rate sensitive index m can be expressed as the derivative ratio of the dissipation co-quantity J and the dissipation quantity G, as shown in Equation (3) [38]:

(3) $$m=\frac{dJ}{dG}=\frac{\partial d\sigma }{\partial d\dot{\varepsilon }}=|\frac{\partial \left(ln\sigma \right)}{\partial \left(ln\dot{\varepsilon }\right)}{|}_{\;T,\varepsilon }$$

Under ideal linear dissipative conditions or when m = 1, the power dissipation coefficient J attains its maximum value, $J_{max}$. The formula is given according to the Prasad criterion definition, and the power dissipation coefficient η can be expressed as follows [37]:

(4) $$\;\eta \;=\;\frac{2m}{m+1}$$

According to Ziegler’s principle of irreversibility in thermodynamics for large plastic flow, Prasad formulated a criterion for identifying plastic deformation instability in materials. This criterion assumes that the parameter m in the dynamic constitutive equation remains unchanged. The instability criterion for rheological behavior is as follows [37]:

(5) $$\;\xi_{p}(\dot{\varepsilon },T)=\;\frac{\partial \ln\left(\frac{m}{m+1}\right)}{\partial \ln\dot{\varepsilon }}+m\;<0$$

**Figure 5:**
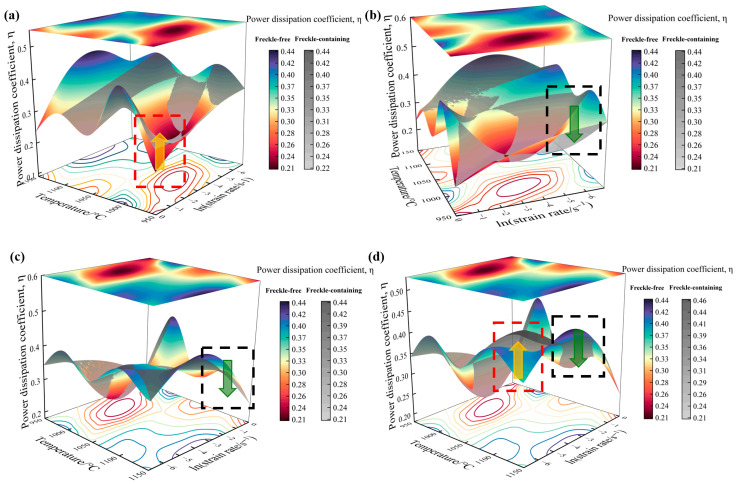
3D comparative analysis of the power dissipation coefficient of the GH4706 alloy freckle-containing specimens and freckle-free specimens under different deformation conditions: (**a**) Dynamic recrystallization zone not initiated; (**b**) dynamic recrystallization zone initiation commenced; (**c**) partial dynamic recrystallization zone; (**d**) dynamic recrystallization zone. The spatial distribution of the η value of the freckle-free specimen is characterized by a 3D color surface. The gray 3D diagram shows the spatial distribution of the power dissipation coefficient η of the specimen with freckles under different deformation conditions. The boxes in the figure indicate the main areas of change, and the arrows indicate the trend of change.

Using Equation (3), the stress and strain values of the modified thermocompression flow stress curve are calculated to obtain the m value at the deformation temperature and strain rate, and then the power dissipation coefficient η of the samples in different deformation regions is calculated using Equation (4). Statistically sort out η values, use a multi-dimensional data visualization method, and use Origin software to draw a 3D network distribution map of η values, as shown in Figure 5. A multidimensional data visualization method was employed to depict the spatial distribution of η values for freckle-free specimens using a colored three-dimensional surface. The top-surface projection displays two-dimensional response characteristics for different orientations, while the bottom-surface contour lines clearly illustrate the gradient pattern of η values. A gray three-dimensional surface depicts the spatial distribution of the power dissipation coefficient η in a freckle-containing pattern under different deformation conditions. The three-dimensional distribution maps of η with and without freckles are superimposed on each other in the same space, which facilitates the comparison of the fine change trends between the two. Comparative analysis reveals that, under low-temperature, high-strain-rate conditions (deformation temperature 950 °C, strain rate 1 s^−1^), η values for freckle-containing specimens are higher than those for freckle-free specimens. Conversely, under conditions of initiated DRX and partial DRX, η values for freckle-containing specimens are approximately 16% lower than those without. At a deformation temperature of 1150 °C, η values within the dynamic recrystallization zone show that freckle-containing specimens have higher η values than those without freckles as the strain rate decreases. Notably, the η value of the freckle-containing specimen increases by 22% to 46% at high temperature and low strain rate (deformation temperature 1150 °C, strain rate 0.001 s^−1^). The η value of the freckle-containing specimen decreases by about 5–10% compared to the freckle-free specimen under the other three conditions. The η value of the freckle-containing specimens remains higher than that of the freckle-free specimens under conditions of high strain rate or high deformation temperature. This indicates that freckle defects significantly affect the deformation mechanism.

**Figure 6:**
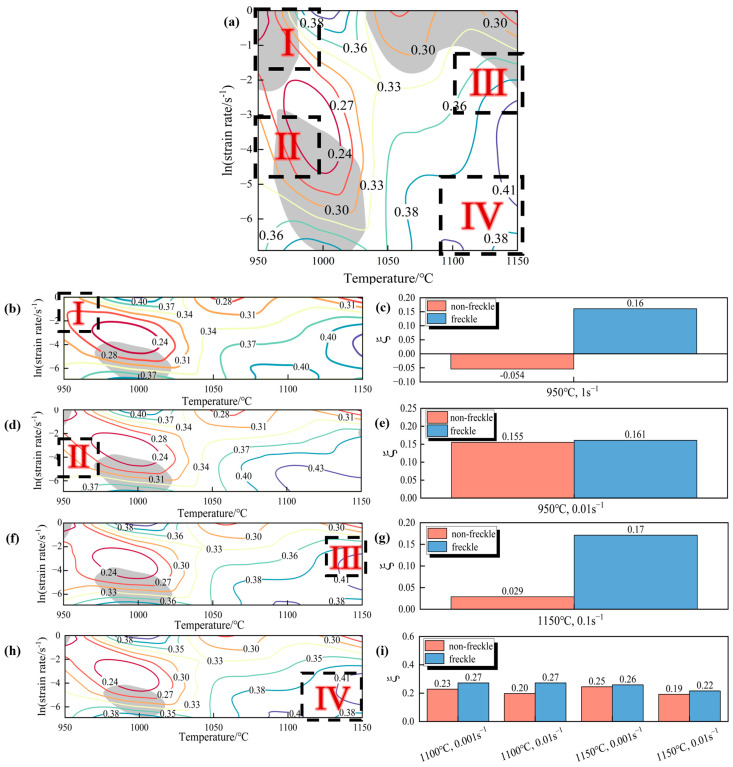
Hot processing maps and instability values ξ; (**a**) hot processing maps of freckle-free specimens; (**b**,**d**,**f**,**h**) hot processing maps of freckle-containing specimens; (**c**,**e**,**g**,**i**) instability values ξ of freckle-containing specimens: (**b**,**c**) 950 °C, 1 s^−1^; (**d**,**e**) 950 °C, 0.01 s^−1^; (**f**,**g**) 1150 °C, 0.1 s^−1^; (**h**,**i**) 1100–1150 °C, 0.001–0.01 s^−1^. The four typical regions (I, II, III, IV) of freckle-free specimens’ hot processing maps can be compared with freckle-containing specimens, where the contour lines in the hot processing maps represent the power dissipation coefficient η value, and the gray shaded area represents the instability value interval (ξ < 0). The different colored lines in the figure represent the variation of the η value in the contour map, and the area selected by the black box is the region for comparing the ξ values of specimens with and without black spots.

According to the η values calculated in Equation (4), the corresponding instability parameters under different conditions can be calculated in Equation (5), and the power dissipation parameters and instability parameters are superimposed and analyzed in the Origin software, and the thermal processing diagram with and without freckles is drawn. As shown in Figure 6, the hot processing maps of GH4706 alloy’s freckle-free specimens are divided into four zones (I, II, III, and IV) for comparison with those of freckle-containing specimens. The contour lines on the maps show the power dissipation coefficient (η), and the gray-shaded areas indicate the range of instability values (ξ < 0). The instability coefficient ξ was calculated under various deformation conditions using Equation (5) from Prasad’s instability criterion. The comparison revealed that the instability coefficient ξ was significantly higher in freckle-containing specimens than in freckle-free specimens, especially at low temperatures with high strain rates (deformation at 950 °C, strain rate 1 s^−1^) and at high temperatures with high strain rates (deformation at 1150 °C, strain rate 0.1 s^−1^). Microstructural optimization processes, such as DRX, contributed to energy dissipation in freckle-containing specimens. Conversely, localized damage, such as adiabatic shear bands and localized flow, was the leading cause in freckle-free specimens. This indicates that during thermal deformation, freckle defects uniquely influence microstructural evolution.

## 4. Effect of Freckle on Microstructural Evolution in GH4706 Alloy

### 4.1. Influence of Freckle on Microstructural Evolution in Non-Dynamic Recrystallization Zones

**Figure 7:**
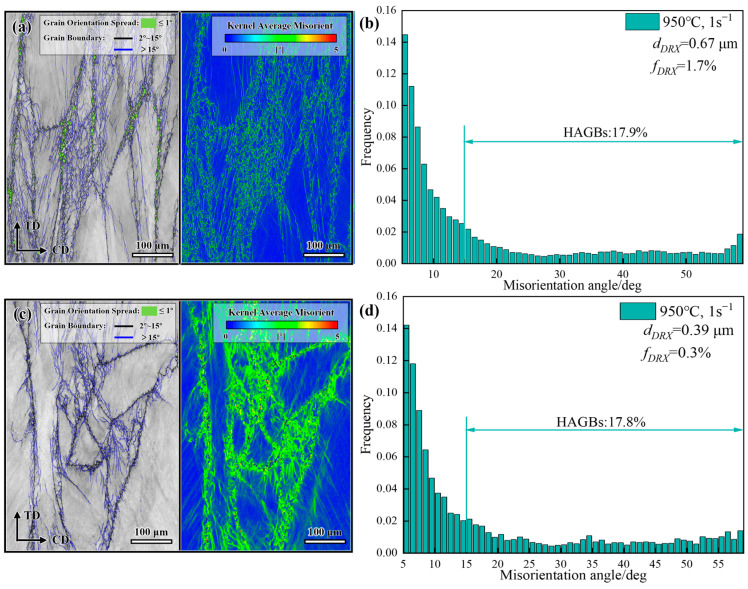
GOS + GB, KAM, and misorientation angle distribution maps of GH4706 alloy at a deformation temperature of 950 °C and a strain rate of 1 s^−1^: (**a**,**b**) freckle-containing specimens; (**c**,**d**) freckle-free specimens.

The DRX evolution of GH4706 alloy was examined at a strain rate of 1 s^−1^ and a deformation temperature of 950 °C using EBSD microstructural analysis (Figure 7).

During plastic deformation, freckle-containing specimens exhibited clear DRX, with high-angle grain boundaries (HAGBs) making up 18% of the overall grain structure. This indicates a high degree of dislocation pile-up at low temperatures and high strain rates. Micrometer-sized DRX grains nucleated within HAGBs, even though significant dislocation buildup usually correlates with strain-hardening. Compared with freckle-free specimens, the freckle-containing specimens exhibited a 20% increase in the DRX volume fraction. The average recrystallization grain size was 0.67 μm, representing a 42% refinement compared to the freckle-free specimens. Along with KAM map analysis, the HAGBs in the freckle-free specimens show a distinct orientation gradient, with an average KAM value of 4.94. This indicates a high dislocation density and notable local strain concentration, placing the material in the rheological instability region. At this stage, local flow and adiabatic shear bands become the dominant deformation mechanisms. Conversely, the KAM value for the freckle-containing specimen decreases to 4.84, indicating greater dislocation annihilation and stress relaxation.

The elements Nb, Ti, and Ni, enriched in freckles, have been shown to accelerate substructure formation and dislocation rearrangement, preferentially initiate the DDRX mechanism, and lower the alloy’s dislocation energy below the critical threshold. This promotes the activation of dislocation cross-slips and climb mechanisms [39]. Variations in recrystallization behavior caused by micro-area compositional segregation directly influence the mechanisms of microstructural development and the flow-stress response during high-temperature deformation of the alloy [38].

**Figure 8:**
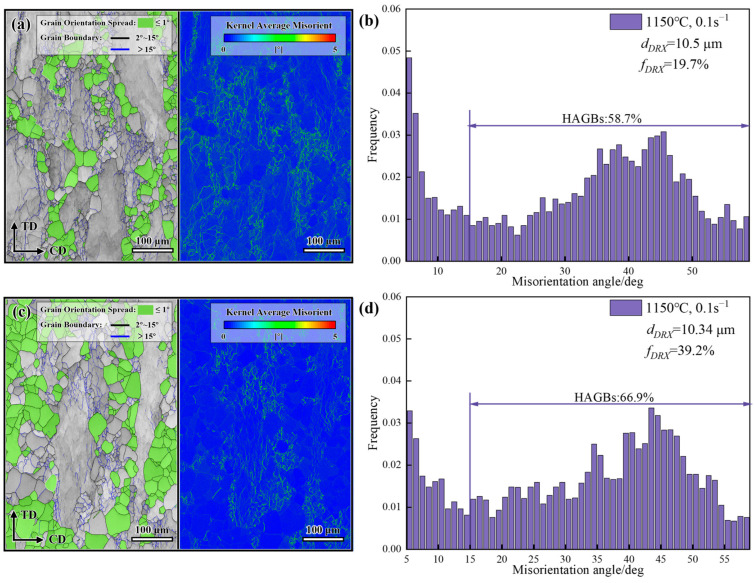
GOS + GB, KAM, and angle diagram of the misorientation maps of GH4706 alloy at a deformation temperature of 1150 °C and a strain rate of 0.1 s^−1^: (**a**,**b**) freckle-containing specimens; (**c**,**d**) freckle-free specimens.

At a deformation temperature of 1150 °C and a strain rate of 0.1 s^−1^ (partially within the dynamic recrystallization zone), Figure 8 shows the hot deformation behavior of the GH4706 alloy.

The freckle-containing specimens exhibited significantly less DRX than the freckle-free specimens. While the HAGBs of the freckle-free specimens displayed dynamically recrystallized grains with uniform sizes, the HAGBs of the freckle-containing specimens showed numerous small sub-grains. KAM analysis revealed transparent orientation gradients within the deformed grains of the freckle-free specimen, with an average KAM value of 4.72. However, due to sufficient DRX, the new grains, free of distortion, absorb dislocations, reducing overall stress concentrations. On the other hand, freckle-containing specimens are in the initial stages of DDRX. At this stage, sub-grains have formed but have not fully transformed into new grains, resulting in low recrystallization. The KAM value of 4.9 indicates a high concentration of dislocation clusters, suggesting that DRX is still incomplete. At 1150 °C, localized Nb segregation promotes DDRX nucleation by lowering the stacking fault energy of dislocation slip in the matrix. Simultaneously, it causes grain boundary pinning effects and dislocation proliferation. This competitive mechanism inhibits DDRX, leading to lower DDRX levels in freckle-containing specimens compared with freckle-free specimens. The continuous increase in dislocation density at sub-grain boundaries leads to a steeper crystal orientation gradient, resulting in an abnormally high KAM value.

### 4.2. Influence of Freckle on Microstructural Evolution in the Dynamic Recrystallization Zone

**Figure 9:**
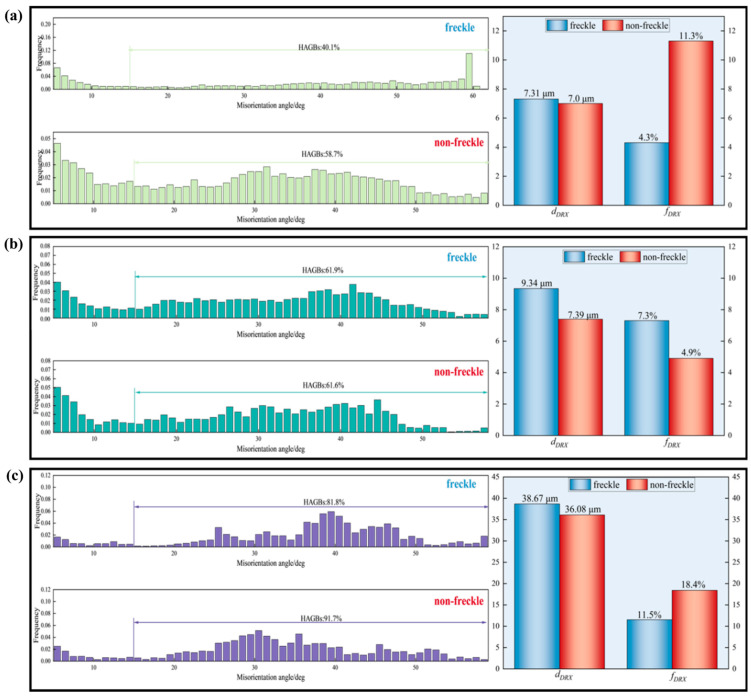
Angle diagram of the misorientation of GH4706 alloy under different deformation conditions: (**a**) 1100 °C/0.01 s^−1^; (**b**) 1150 °C/0.01 s^−1^; (**c**) 1150 °C/0.001 s^−1^. On the left is the bar diagram of misorientation angle and frequency of the two specimens, and on the right is the column comparison diagram of f_DRX_ (recrystallized grain volume) and d_DRX_ (average size of recrystallized grains).

Under deformation conditions of 1100–1150 °C for GH4706 alloy and strain rates of 0.001–0.01 s^−1^, the differences between freckle-containing specimens and freckle-free specimens were quantified after deformation. The analysis results are shown in Figure 9.

At a deformation temperature of 1100 °C and a strain rate of 0.01 s^−1^, the DRX volume fraction of the freckle-containing specimen decreased significantly by 62%. The average size of the DRX grains in the freckle-containing specimens was larger than in the freckle-free specimens. Interestingly, at a strain rate of 0.01 s^−1^ and a temperature of 1150 °C, the HAGBs and dynamic recrystallized volume fractions were similar. However, in the freckle-containing specimen, the average size of the dynamic recrystallized grains increased by 21%, reaching 9.34 μm. This is due to DRX lag in freckle-containing specimens, which leads to greater strain energy storage during the low-temperature deformation stage. Higher temperatures promote grain coalescence and growth by releasing stored energy and accelerating grain boundary movement [40]. At 1150 °C and a strain rate of 0.001 s^−1^, the freckle-containing specimen had a larger average grain size compared to the freckle-free specimens. Both specimens experienced significant grain coarsening. The DRX size of the freckle-containing specimen was 38.67 μm, larger than the average size of the recrystallized grains in the freckle-free specimens. Increasing the temperature to 1150 °C and lowering the strain rate to 0.001 s^−1^ extended the deformation time, allowing the high-density dislocations in the initial stages of freckle-containing specimens to be more fully released through dynamic recovery (DRV) and recrystallization. The stored strain energy is converted into a driving force for grain boundary migration, further accelerating grain boundary movement and promoting rapid recrystallization, with grains engulfing adjacent sub-grains and small grains and encouraging DRX of grain growth [41,42].

Quantitative investigation indicates that freckle defects at strain rates of 0.001–0.01 s^−1^ and deformation temperatures of 1100–1150 °C significantly influence the microstructural evolution of GH4706 alloy within the DRX region. Compared to freckle-free specimens, HAGBs are more prevalent in freckle-containing specimens. The percentage of DRX volume and the average grain size are larger than those in freckle-free specimens.

### 4.3. Influence of Freckle on the Dynamic Recrystallization Mechanism

#### 4.3.1. Non-Dynamic Recrystallization Zones

**Figure 10:**
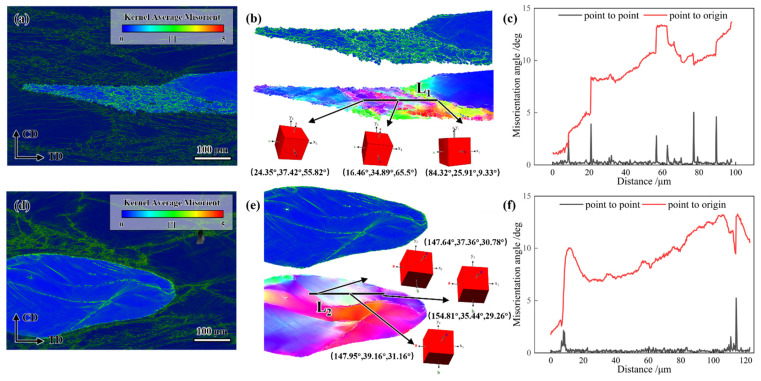
KAM maps, high-magnification micrograph, and IPF maps of GH4706 alloy under a deformation temperature of 950 °C and a strain rate of 1 s^−1^: (**a**–**c**) freckle-containing specimens; (**d**–**f**) freckle-free specimens. The change in grain orientation difference in the underlined line in (**b**,**e**) corresponds to the shift in (**c**,**f**). The arrows at the underlined lines L_1_ and L_2_ point to the respective cubic blocks representing the grain orientations.

The impact of freckle defects on the DRX mechanism of the GH4706 alloy after hot deformation is qualitatively analyzed and presented in Figure 10. At a deformation temperature of 950 °C and a strain rate of 1 s^−1^ (without initiating the DRX region), the cumulative orientation difference along the L_1_ black line within the deformed grains of the freckle-containing specimen did not exceed 15° [43], as shown in Figure 10c. The original grain boundaries were covered mainly by multiple fine, equiaxed DRX grains, forming a necklace structure, while some HAGBs displayed serrate patterns. The primary DRX mechanism is DDRX, as evidenced by the necklace-like DRX grains and serrated grain boundaries. The initial grain boundaries protrude due to strain during nucleation. DDRX dominates the DRX process. In contrast, local flow is the primary deformation mechanism in the freckle-free specimen, which has not begun DRX.

**Figure 11:**
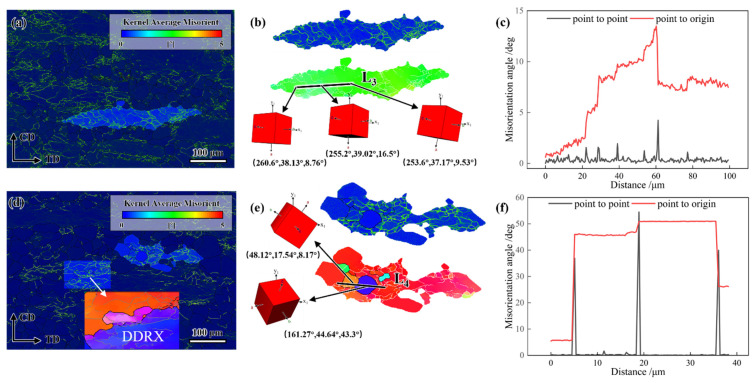
KAM map, high-magnification micrograph, orientation distribution map, and misorientation angle variation along the marked black line of GH4706 alloy deformed at 1150 °C/0.1 s^−1^: (**a**–**c**) freckle-containing specimens; (**d**–**f**) freckle-free specimens. The change in grain orientation difference in the underlined line in (**b**,**e**) corresponds to the shift in (**c**,**f**). The arrows at the underlined lines L_3_ and L_4_ point to the respective cubic blocks representing the grain orientations.

To qualitatively analyze the influence of freckle defects on the DRX mechanism alloy after hot deformation, the results are presented in Figure 11.

At a deformation temperature of 1150 °C and a strain rate of 1 s^−1^, freckle-containing specimens exhibited DDRX as the primary DRX mechanism. Within the distorted grains, the cumulative orientation difference along the L_3_ black line was less than 15°. Conversely, under high-temperature or low-strain-rate hot deformation conditions, freckle-free specimens tended to undergo CDRX-mediated microstructural evolution, characterized by progressive grain boundary migration dominated by subgrain coalescence. High dislocation density was indicated by the cumulative orientation difference along the L_4_ black lines within distorted grains, which exceeded 15°. Sub-grain rotation produced new dynamic recrystallized grains due to further deformation. Notable variations in 3D unit-cell orientations were observed in Figure 11b, further supporting the CDRX mechanism. Interestingly, the HAGBs display a sawtooth pattern during expansion, typical of the DDRX mechanism, at the large-angle grain boundaries of the freckle-free specimens. Therefore, CDRX and DDRX serve as the nucleation processes in freckle-free specimens.

#### 4.3.2. Dynamic Recrystallization Zone

**Figure 12:**
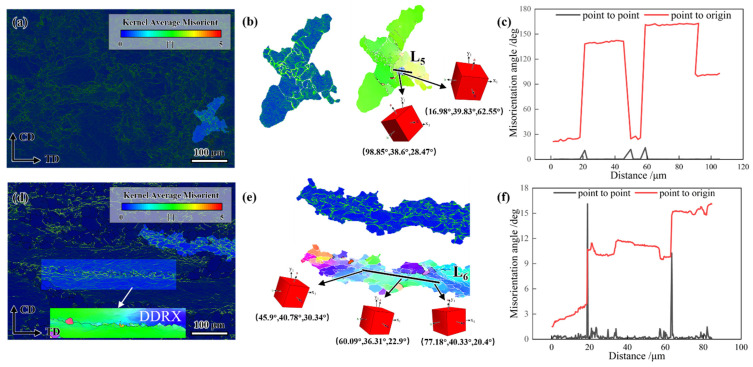
KAM map, high-magnification micrograph, orientation distribution map, and misorientation angle variation along the marked black line of GH4706 alloy deformed at 1100 °C/0.01 s^−1^: (**a**–**c**) freckle-containing specimens; (**d**–**f**) freckle-free specimens. The change in grain orientation difference in the underlined line in (**b**,**e**) corresponds to a shift in (**c**,**f**). The arrows at the underlined lines L_5_ and L_6_ point to the respective cubic blocks representing the grain orientations.

KAM and IPF maps of GH4706 alloy specimens with and without freckle defects at a deformation temperature of 1100 °C and a strain rate of 0.01 s^−1^ are shown in Figure 12.

Local orientation variations of over 15° along the L_5_ black line are observed in freckle-containing specimens within deformed grains, indicating that CDRX is the primary DRX mechanism. Under high-temperature or low-strain-rate hot deformation conditions, the alloy tends to evolve microstructures via the CDRX process, characterized by progressive grain boundary migration dominated by subgrain coalescence. Significant differences in 3D unit-cell orientations confirm the CDRX process. The freckle-free specimens exhibit HAGBs with serrated features. The development of these serrated borders suggests that DDRX is the primary DRX mechanism. The local protrusion effect of the initial grain boundaries under strain is responsible for its nucleation mechanism. Combined with KAM images, dislocations primarily pile up near HAGBs, further validating the DDRX-dominated DRX mechanism. An asymptotic rotation tendency of sub-grains was indicated by the local orientation deviation along the L6 black line exceeding 15° within damaged grains. New DRX grains formed by sub-grain rotation as deformation progressed. Figure 12b revealed significant differences in 3D unit cell orientations, confirming the CDRX mechanism as the primary DRX pathway.

**Figure 13:**
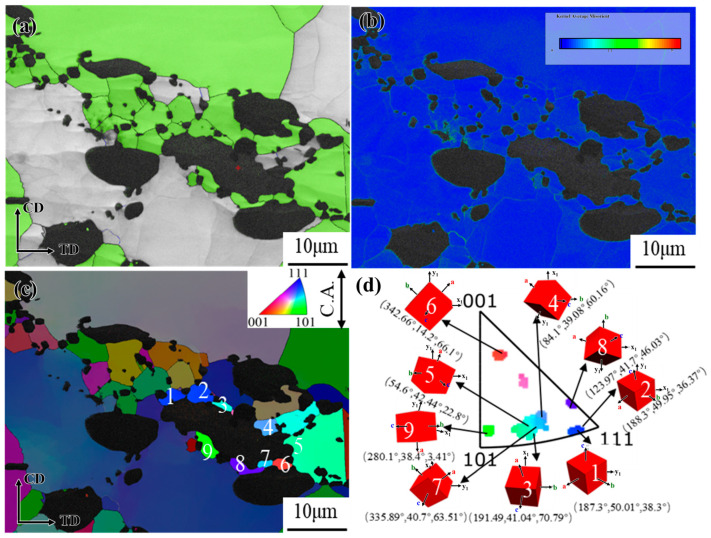
GOS, KAM, and IPF maps of GH4706 alloy freckle-containing specimens after deformation at 1100 °C/0.001 s^−1^: (**a**) GOS map; (**b**) KAM map; (**c**,**d**) IPF map. The black area is the freckle, and the grain orientation of the reverse polar diagram in (**d**) and the corresponding grain orientation squares 1–9 correspond to correspond to the grains 1–9 near the marked freckles in (**c**).

Microstructural analysis of GH4706 alloy with freckle defects after deformation at 1100 °C and 0.001 s^−1^ using EBSD characterization (Figure 13) shows that numerous fine, equiaxed grains surround small phase particles (5–10 μm) within the freckle defects, which precipitate on the HAGBs. Coupled with GOS mapping, the orientation gradient within these grains is nearly zero, indicating the grains are newly formed DRX grains. It implies that the freckle defects’ micro-sized precipitates act as nucleation sites for DRX grain formation. Significantly less dislocation pileup was seen in DRX grains located farther away from the precipitates, whereas prominent dislocation pileup was seen in the vicinity of the precipitates. DRX grain nucleation was also observed around large precipitates, yet grain sizes (5–10 μm) were 60% smaller than those around smaller precipitates. Since freckle defects reconfigure the thermodynamic and kinetic conditions for DRX through composition-phase coupling, DDRX emerges as the dominant mechanism, exhibiting a typical PSN mechanism. Compared with the freckle-free specimens, the recrystallized grains around the freckles improve the overall structure inhomogeneity, which may also have a significant impact on the subsequent durability, high-temperature tensile, and fatigue properties of the alloy [17]. In addition, during the aging process of the alloy after thermal deformation, there is not only the inhomogeneity of grain size in the freckle area, but also the elemental segregation around the freckles and the grain size change during the thermal compression process, which leads to the formation of partially ordered reinforcing phases (γ′, γ″) around the freckles during the aging process, and the distribution of such reinforcing phases will also have a profound impact on the subsequent mechanical properties of the alloy.

### 4.4. The Fundamental Reason Why Freckles Induce Differences in the Mechanism of Dynamic Recrystallization

**Figure 14:**
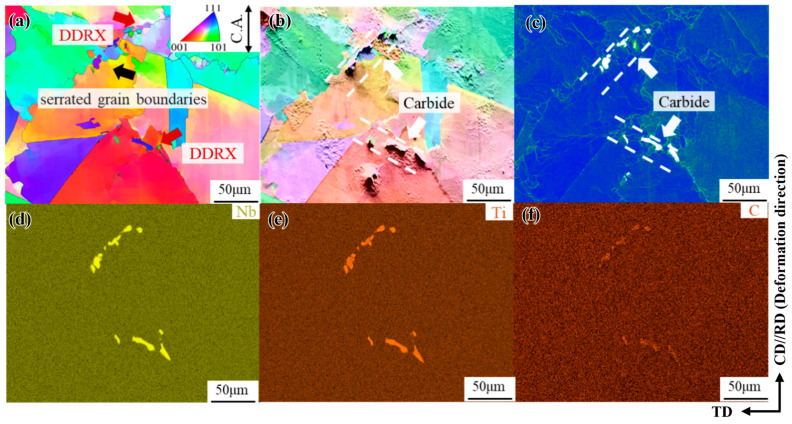
Microstructure and composition diagram of GH4706 alloy freckle-containing specimens under 1100 °C/0.01 s^−1^: (**a**) IPF map; (**b**) EBSD layered map; (**c**) KAM map; (**d**–**f**) Nb, Ti, and C energy dispersive spectroscopy (EDS) spectra. The arrow points to the carbide. In the figure, the red arrow points to the necklace-like structure, which is a typical DDRX mechanism feature, and the black arrow points to the jagged grain boundary.

The influence of carbides is significant under conditions of 1100 °C and a strain rate of 0.01 s^−1^. The IPF diagram, KAM image, and EDS spectrum of the freckle-containing specimen are illustrated in Figure 14. The recrystallization mechanism of the GH4706 alloy varies significantly with strain rate. As the strain rate increases, it creates favorable energy and structural conditions, promoting the storage of deformation energy and the formation of recrystallized grains. Large-angle grain boundaries display serrated profiles, where differences in dislocation gradients at the boundaries encourage bowing nucleation of primary grain boundaries. This process is characteristic of DDRX, which results in grain boundary expansion. Additionally, small carbides enriched with titanium (Ti), carbon (C), and niobium (Nb) tend to precipitate along the grain boundaries, providing nucleation sites for DRX [44,45]. This incorporation of DRX processes helps reduce local stress.

**Figure 15:**
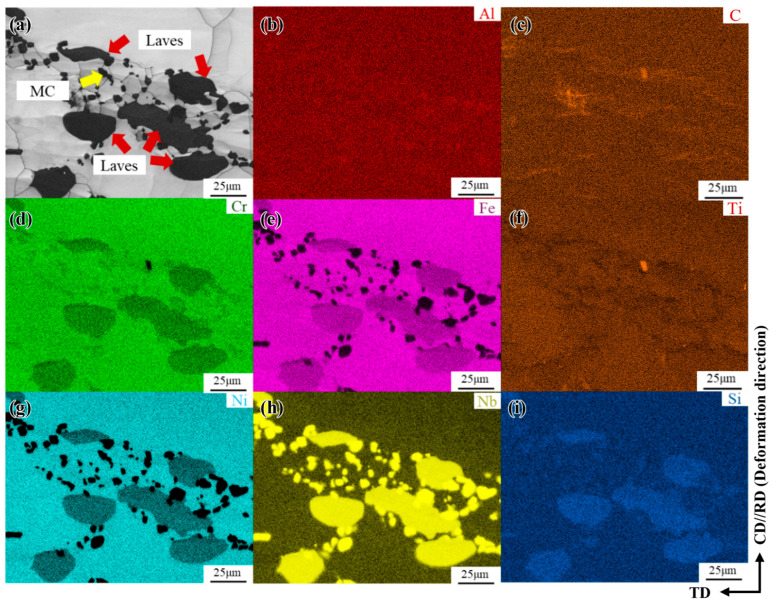
BC and EDS spectra of freckle-containing specimens of GH4706 alloy under 1100 °C/0.001 s^−1^: (**a**) morphology diagram; (**b**–**i**) Al, C, Cr, Fe, Ti, Ni, Nb, and Si energy dispersive spectroscopy (EDS) spectra. The yellow arrow points to the carbide, and the red arrow points to the Laves phase, which is more densely precipitated and sandwiched between the larger Laves phases.

The second factor is the influence of the Laves phase. Current research indicates [46,47,48,49] that the GH4169 alloy employs a homogenization treatment process of “1130 °C, 24 h + 1160 °C, 24 h + 1190 °C, 48 h” to eliminate the Laves phase. This achieves uniform distribution of the primary segregation element Nb, thereby removing the Laves phase. As illustrated in Figure 15, granular Laves phase precipitates remain undissolved at grain boundaries after hot deformation at 1100 °C with a strain rate of 0.001 s^−1^, displaying considerable stress concentration. Moreover, no local orientation gradient is observed within grains, indicating near-complete DRX. PSN was suggested by the presence of some recrystallized grains close to Laves phase particles, especially in grain boundary locations with sizable particle clusters (Figure 15). Because of strain incompatibility between coarse Laves phase particles and the matrix, such particles may cause non-uniform deformation around themselves. As shown in the KAM map in Figure 14, these particle-associated deformation bands become favorable nucleation sites for recrystallized grains during hot deformation [50]. In EBSD microstructures, GOS is used to differentiate between recrystallized and unrecrystallized parent-phase grains. Grains with GOS ≤ 1° are considered recrystallized grains, while those with GOS > 1° are regarded as unrecrystallized grains [51,52,53]. The GOS and KAM maps in Figure 14 reveal that a small fraction of recrystallized grains exhibit virtually no low-angle grain boundaries (LAGBs) or sub-structures internally. This indicates that the DRX process is nearly complete and that the Laves phase does not inhibit it.

The influence of the η phase ranks third. There were no signs of the η phase in the IPF diagrams after thermal deformation under different settings. At 1100 °C and a strain rate of 0.001 s^−1^, the freckle-containing specimens showed a higher DRX volume percentage than the freckle-free specimens. Recent research indicates that η-phase dissolution begins at 980 °C and ends at 1100 °C. According to Liu [54], dislocations formed during thermal deformation tend to promote DRX behavior through sub-grain rotation nucleation, as such dislocations mainly gather at η phase boundaries. The η phase becomes more susceptible to deformation and fracture due to the torsional stresses caused by these sub-grain rotations and the development of DRX grains. High-density dislocations accumulate at these interfaces and act as diffusion pathways for solute elements around the η phase, speeding up the dissolution of the η phase. This is because the DRX temperature is close to the precipitation temperature of the η phase (950 °C). Nonetheless, a residual η phase remains when the alloy deforms at temperatures just below twinning dynamic recrystallization (TDRX), preventing sub-grain or grain boundary migration [55,56,57]. Increasing deformation temperature encourages either dislocation buildup at η-phase boundaries or η-phase dissolution. During DRV, LAGBs transform into HAGBs, forming DRX grains.

**Figure 16:**
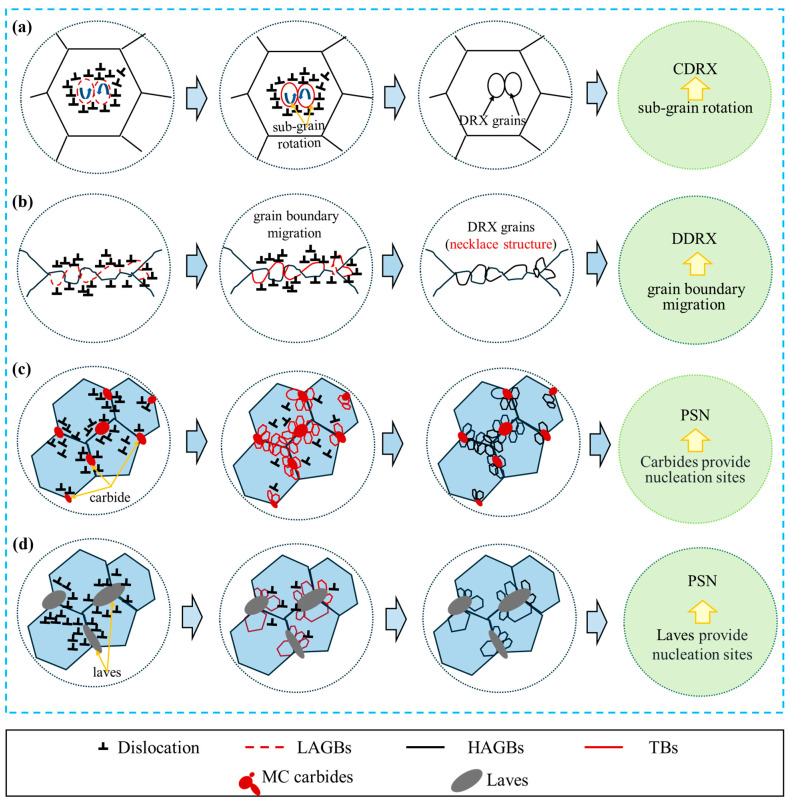
Diagram of the dynamic recrystallization mechanism of GH4706 alloy after hot deformation: (**a**) continuous dynamic recrystallization; (**b**) discontinuous dynamic recrystallization; (**c**) particle excitation nucleation mechanism of MC carbides; (**d**) particle excitation nucleation mechanism of Laves phase.

Two distinct DRX mechanisms were identified in the GH4706 alloy through multidimensional EBSD analysis of the microstructure after thermal deformation: DDRX and CDRX. Figure 16 illustrates the DRX mechanisms, where Figure 16a represents CDRX, Figure 16b shows DDRX, and Figure 16c,d illustrate DRX mechanisms triggered by second-phase particles.

At low strain rates or high deformation temperatures, DRV can consume dislocations rapidly in alloy materials. Consequently, the dislocation density at grain boundaries may be too low to cause the original boundaries to bulge. Through DRV, dislocation cells can form near the initial grain boundaries and then transform into LAGBs. These LAGBs are subsequently converted into HAGBs via sub-grain rotation, resulting in the formation of new CDRX grains. To promote the development of additional CDRX grains, primary grain boundaries may migrate during or after the formation of these new CDRX grains. The process of CDRX grain formation within a primary grain is shown in Figure 16a. Dislocation cells may form within the prominent grain due to DRV and then split into sub-grains. During further deformation, sub-grains can transform into new DRX grains through sub-grain rotation.

Grain boundaries can hinder dislocation motion, as shown in Figure 16b, thereby increasing dislocation density near the boundaries. This indicates higher stored energy. Grain boundary expansion occurs when the stored energy pushes boundaries toward regions with high dislocation density. As dislocations continue to accumulate during further deformation, DRV leads to the formation of dislocation cells and LAGBs in sequence. Sub-grains then develop between the projecting grain borders and the LAGBs. These sub-grains continuously absorb dislocations and gradually convert into HAGBs, ultimately forming a small DDRX grain. Migration of the grain boundary can promote further growth of this grain. The formation of several tiny DDRX grains along the original grain boundaries creates a necklace-like structure.

DRX is frequently observed near MC carbides, as shown in Figure 16c,d. According to EBSD studies, MC carbides serve as nucleation sites for particle-induced DRX. Simultaneously, particle-stimulated nucleation occurs during the initiation of recrystallization at boundaries containing Laves phases. In these cases, Laves phase particles serve as nucleation sites, triggering the PSN mechanism. Notably, the original HAGBs are tightly encased by fine, equiaxed DRX grains, resulting from the combined action of these two nucleation mechanisms and forming a distinctive “necklace structure.” The spatial continuity of this structure and the abrupt changes in grain boundary migration paths align with DDRX’s “nucleation-annexation” evolution pattern.

## 5. Conclusions

1. MC carbides are concentrated in the vicinity of Nb-rich Laves phases and Ti-rich η phases, which severely segregate and coarsen to generate the microstructural features of the freckle region.
2. Under identical deformation conditions, compared to the flow curve trend of freckle-free specimens, the flow curve trend of freckle-containing specimens initially follows a higher trajectory before subsequently falling below. Peak stress shows apparent differences: freckle-containing specimens have higher peak stress than freckle-free specimens at deformation temperatures of 1100–1150 °C and strain rates of 0.01 s^−1^, while the opposite is true under other conditions.
3. The instability zone of freckle-containing specimens significantly decreased when combined with diagram analysis during thermal processing. Microstructural optimization driven by DRX primarily governed energy dissipation in both low-temperature, high-rate, and high-temperature, low-rate conditions, as indicated by higher η values and an instability value ξ > 0. Under other conditions, freckle-containing specimens exhibited lower η values compared to those without, with insignificant changes in ξ, suggesting similar energy-dissipation mechanisms.
4. In non-dynamic recrystallization zones, DRX grains in freckle-containing specimens formed around deformed grains. The level of DRX in freckle-containing specimens was significantly lower than in freckle-free specimens as the deformation temperature increased. The proportion of HAGBs in freckle-containing specimens was lower than in freckle-free specimens within the dynamic recrystallization zone. While the percentage of DRX volume showed complex fluctuations, the average size of dynamic recrystallized grains in freckle-containing specimens was larger than in freckle-free specimens. Grain coarsening was observed in freckle-containing specimens, with the average dynamically recrystallized grain size reaching 38.6 μm under high-temperature, low-strain-rate conditions.
5. DRX mechanisms involving DDRX and CDRX were demonstrated in both freckle-containing and freckle-free specimens. Grain-boundary migration, subgrain rotation, and expansion all contribute to DDRX. In the DDRX mechanism, nucleation sites at HAGBs are supplied by MC carbides and Laves phases within the freckle, displaying a typical PSN mechanism. CDRX can occur via subgrain rotation or a combination of subgrain rotation and grain boundary migration.

## Data Availability

The original contributions presented in this study are included in the article. Further inquiries can be directed to the corresponding author.
